# Free-Energy-Based Design Policy for Robust Network Control against Environmental Fluctuation

**DOI:** 10.1155/2015/464031

**Published:** 2015-06-08

**Authors:** Takuya Iwai, Daichi Kominami, Masayuki Murata, Tetsuya Yomo

**Affiliations:** ^1^Graduate School of Information Science and Technology, Osaka University, Suita, Osaka 565-0871, Japan; ^2^Graduate School of Economics, Osaka University, Toyonaka, Osaka 560-0043, Japan

## Abstract

Bioinspired network control is a promising approach for realizing robust network controls. It relies on a probabilistic mechanism composed of positive and negative feedback that allows the system to eventually stabilize on the best solution. When the best solution fails due to environmental fluctuation, the system cannot keep its function until the system finds another solution again. To prevent the temporal loss of the function, the system should prepare some solution candidates and stochastically select available one from them. However, most bioinspired network controls are not designed with this issue in mind. In this paper, we propose a thermodynamics-based design policy that allows systems to retain an appropriate degree of randomness depending on the degree of environmental fluctuation, which prepares the system for the occurrence of environmental fluctuation. Furthermore, we verify the design policy by using an attractor selection model-based multipath routing to run simulation experiments.

## 1. Introduction

For information and communication networks to serve as an indispensable part of the infrastructure for secure, dependable, and comfortable society, they must be more robust against ever-increasing size, dynamic changes, and complexity [1]. In recent years, many researchers have focused on interdisciplinary approaches to spark innovative ideas. In particular, they have been actively working on network controls that are inspired by biological behavior, and many published papers support the usefulness of such systems [2–4].

A bioinspired network control relies on a probabilistic mechanism composed of positive and negative feedback that allows searching for better solutions. On finding better solutions, the system more aggressively selects in the direction of those solutions as a result of its positive feedback. The system eventually stabilizes on the best solution by minimizing its negative feedback [5]. This type of mechanism will need to search for a solution again in the case where the known solution fails due to environmental fluctuations, such as node failures and link failures. Therefore, it is not possible to avoid a temporary loss of function. Here we describe a simple example taking wireless sensor networks (WSN) [6]. A WSN consists of a large number of wireless communication sensors, which send their sensing data to sinks in a multihop manner. Wireless communication is quite sensitive to environmental noise. An optimum route from a sensor to a sink at a certain time can become unavailable after a while, and the sink fails in gathering a part of sensing data from the sensor until the network adapts its internal settings such as routing information to the latest environment. These imply that it is not always reasonable for the network control to select the solution that is optimum at a particular time. To prepare for the occurrence of environmental fluctuation, it is quite important that such systems also select other solutions in addition to the optimum one. However, most existing bioinspired network controls are not designed with this issue in mind.

Let us assume that the tendency for bioinspired network controls to select the optimum solution is measured by* ordering energy*. The ordering energy relates to their potential performance, which we can potentially obtain in environments that have no fluctuation. We also assume that the tendency for them to select other solutions in addition to the optimum solution is measured by* disordering energy*. Disordering energy is related to performance degradation caused by environmental fluctuation [7]. Using ordering energy and disordering energy, we can describe an appropriate design for the steady state. When a system is deployed in a stable environment, its steady state should be designed to have high ordering energy. In contrast, for systems in dynamic environments, the design should prevent its performance from degrading and therefore should cause the steady state to have low disordering energy [8]. As a consequence, we need to design systems to balance between their ordering energy and their disordering energy according to the degree of environmental fluctuation as a means of preparing the systems for the occurrence of environmental fluctuation.

To give a quantitative interpretation of ordering and disordering energy, we focus on thermodynamic free energy, which indicates the state of a natural substance. Thermodynamics says that free energy *A* is formulated by equation *A* = *E* − *T* × *S*, where *A*, *E*, *T*, and *S* are the free energy, internal energy, temperature, and entropy, respectively. The details are described in Section 2, but we describe the model briefly here. Internal energy *E* corresponds to ordering energy. The product *T* × *S* corresponds to disordering energy. Assuming that temperature *T* does not change, this equation implies that the temperature *T* can be used to determine which change is effective in changing free energy *A*, internal energy *E*, or entropy *S*. From the standpoint of designs for bioinspired network controls, we can obtain a design for balancing the ordering energy with the disordering energy by regarding temperature *T* as the degree of environmental fluctuation, which allows us to prepare systems for its occurrence. The obtained design contributes to making performance when the fluctuation occurs higher than other designs.

In this paper, we establish and verify a thermodynamics-based design policy for network controls that will cause them to reach the steady state appropriate to the degree of environmental fluctuation. For this purpose, we first explain a steady state of network controls in terms of thermodynamics. Next we preliminarily investigate an appropriate steady state, which depends on the given degree of environmental fluctuation. Then, we build an analytical network control and formulate and analyze its thermodynamic state values. From the analytical results, we obtain a thermodynamics-based design policy. Finally, we verify the design policy by using an attractor selection model-based multipath routing [9] as an existing control.

The rest of this paper is organized as follows. First, we describe the interpretation of network controls from the perspective of thermodynamics in Section 2. Next, in Section 3, we explain preliminary simulation results. Then, we build and analyze an analytical model of a network control, and we establish a design policy in Section 4. In Section 5, we verify the design policy. In Section 6, we describe related work. Finally, we conclude this paper and remark on future work.

## 2. Thermodynamic Interpretation

By analyzing a network control from the perspective of its free energy, we can discuss the balance between the system's ordering energy and its disordering energy according to the degree of environmental fluctuation. In the following subsections, we briefly introduce a thermodynamic model and interpret the state of a network control from the thermodynamic perspective.

### 2.1. Thermodynamics

Natural substances stabilize in the state with the minimum free energy [10]. Free energy is generally formulated by the following equation:

(1) $$\begin{array}{c} A=E-T\times S, \end{array}$$

where *A*, *E*, *T*, and *S* are the free energy, internal energy, temperature, and entropy, respectively. Collectively, these values are “thermodynamic state values.” Internal energy *E* is the stored energy, which we can potentially extract as heat or work, and corresponds to the ordering energy. Entropy *S* measures the difficulty of extracting energy from the substance. A higher entropy *S* means that it is more difficult to transform the internal energy *E* to heat or work. Temperature *T* measures the average energy of particles of the substance. When the temperature *T* is higher, the substance includes particles with higher energy. From the definitions of temperature *T* and entropy *S*, their product *T* × *S* measures the energy that cannot be extracted from the substance; this corresponds to the disordering energy. From (1), this implies that the free energy *A* is the energy that we can actually extract from the substance. Thus, natural substances become stabilized in a state where change from internal energy to heat or work does not occur. The descriptions above are summarized in Table 1.

### 2.2. Network Controls

A good state (e.g., a routing table with the shortest paths) of network controls emerges via direct or indirect interactions among nodes. Let us assume here that when a network control has higher internal energy *E*_nw_, it can potentially achieve higher performance. Note here that the term “potentially” means in an ideal environment, where no fluctuation occurs. For the sake of simplicity, the internal energy *E*_nw_ is called “optimality.” We assume that a network control has higher entropy *S*_nw_ when its performance is more susceptible to environmental fluctuation. Therefore, the rate of performance degradation for the given degree of fluctuation corresponds to the system's entropy. For the sake of simplicity, entropy *S*_nw_ is called “robustness” (note that in this paper small *S*_nw_ means high robustness). We assume that a network control has a higher temperature *T*_nw_ when the environment changes more frequently. Therefore, we can quantify the temperature by node failure rate, link error rate, and so on. We find that the product *T*_nw_ × *S*_nw_ corresponds to the performance decrease caused by the fluctuation; this follows from the interpretations of *S*_nw_ and *T*_nw_. We also find that free energy *A*_nw_ corresponds to the performance actually achieved in a fluctuating environment, as shown in (1). Thus, we should design network controls to have higher free energy. The interpretations given above are summarized in Table 2.

## 3. Preliminary Investigation

We preliminarily investigate an appropriate design for a network control that accounts for the degree of environmental fluctuation. For this purpose, taking multipath routing [11] as an example of a network control, we conduct simulation experiments to investigate the influence of a design whose optimality and robustness are different components of performance.

### 3.1. Simple Model of a Multipath Network

We here consider a simple model of a multipath network. In this model, many wireless nodes are randomly distributed in the field, and each wireless node can communicate with other wireless nodes that are within a certain radius. Some node-disjoint paths are constructed between a pair consisting of a source node and a destination node.

**Figure 1:**
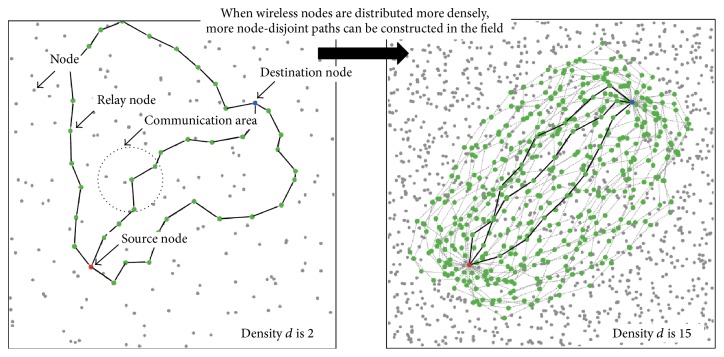
Influence of node density *d* on node-disjoint paths between the source node and the destination node.

More specifically, wireless nodes are randomly distributed in a field of size 10 m × 10 m. To distribute the nodes uniformly, we partition the field into blocks of size 1 m × 1 m and randomly allocate *d*  (≥1) wireless nodes in each of the blocks. The constant *d* is called the “node density.” In total, there are 100 × *d* wireless nodes in the field. In addition to these 100*d* wireless nodes, we place two more wireless nodes, one at (2.5,2.5) and one at (7.5,7.5). We call the former wireless node the “source node” and the latter wireless node the “destination node.” Two examples of this wireless network are illustrated in Figure 1.

Each wireless node can communicate with other wireless nodes whose position is within a circle of radius 1 m. A wireless node succeeds in sending a packet to a neighboring wireless node with probability 1 − *l*_*i*_, where the constant  *l*_*i*_  (0 ≤ *l*_*i*_ ≤ 1) is the probability that a packet to node *i* is dropped due to environmental fluctuation. Let us assume a simple model of environmental fluctuation. In this model, the source of environmental fluctuation is at position (5,5), in the center of the field. Environmental fluctuations affect the probability *l*_*i*_ for wireless node *i* according to the following formula:

(2) $$\begin{array}{c} l_{i}=q_{\max}\times \exp\left[\;-\frac{{\left(x_{i}-5\right)}^{2}+{\left(y_{i}-5\right)}^{2}}{2\times \sigma^{2}}\;\right]. \end{array}$$

In this equation, the position of node *i* is denoted by (*x*_*i*_, *y*_*i*_). The constant *q*_max⁡_  (0 ≤ *q*_max⁡_ ≤ 1) sets the degree of environmental fluctuation. As this constant is larger, the environment more frequently fluctuates. The constant *σ* ^2^  (>0) denotes the extent of the area affected by environmental fluctuation. As the constant *q*_max⁡_ becomes larger, environmental fluctuation occurs more frequently. As the constant *σ* ^2^ becomes larger, a wider area is affected by environmental fluctuation.

We search for all node-disjoint paths between the source node and the destination node. We use the shortest three paths as “path candidates.” When the source node sends a packet to the destination node, it probabilistically selects a path from among the path candidates. The probability that the source node selects path *j* is given by a set of constants *p*_*j*∈{1,2,3}_  (0 ≤ *p*_*j*_ ≤ 1) in advance. A packet is iteratively forwarded to the destination node along the selected path.

### 3.2. Path Candidates with Different Characteristics

We prepare two types of path candidates, which have different characteristics: hop length and susceptibility to environmental fluctuation. For this purpose, we investigate topologies with different node densities *d*.

The left panel of Figure 1 is a sample of node-disjoint paths when nodes are sparsely distributed (*d* = 2). The right is a sample of node-disjoint paths when nodes are densely distributed (*d* = 15). In these figures, black lines denote the path candidates, that is, the three shortest node-disjoint paths. Gray lines denote other node-disjoint paths. Red, blue, and green nodes are the source, destination, and relay nodes, respectively. When the node density *d* is 2, there are a small number of node-disjoint paths. These paths detour, and the path candidates are long paths and cover a wide area. When node density *d* is 15, there are a large number of node-disjoint paths. These paths lie near the shortest path, and here they are as short as the shortest path.

**Figure 2:**
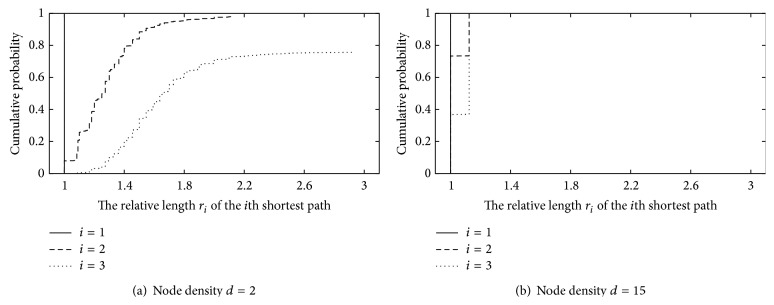
Influence of node density *d* on characteristics of the three shortest node-disjoint paths.

Figure 2 shows the relative lengths *r*_*j*_  (≥1) of the *j* shortest paths. Here the relative length *r*_*j*_ is defined as *r*_*j*_ = *n*_*j*_/*n*_1_. *n*_*j*_  (≥1) is the hop length of the *j*th shortest path. Specifically, *n*_1_ is the hop length of the shortest path. For each node density, we randomly generate 1,000 topologies and calculate *r*_*j*_ for all path candidates and topologies. Figures 2(a) and 2(b) are the cases for node density *d* at 2 and 15, respectively. In these figures, the *x*-axis corresponds to the relative length *r*_*j*_, and the *y*-axis corresponds to the cumulative incidence of successful transmission in the trials.

Figure 2(a) shows that the cumulative probability does not always reach 1 when the node density *d* is 2, that is, in the sparse case. This means that it becomes more difficult to find suitable detours because the wireless nodes are more sparsely distributed. There are at least two successful paths in 980 samples out of 1,000 samples. However, half of them are 1.2 times longer than the shortest one. In the worst case, they are 2.1 times longer. In 757 of 1,000 samples, we can obtain three paths. The middle-length paths are 1.5 times longer than the shortest path. The longest paths are 3.0 times in the worst case. Thus, the number of detours is small in the sparse case. However, the detours lie in a wide area far from the shortest path, and the path candidates are not simultaneously affected by environmental fluctuation.

In contrast, Figure 2(b) shows that the cumulative probability always reaches 1. This result means that detours always exist because nodes are more densely distributed. In 70% of all samples, the hop length of the second-shortest path is equal to the hop length of the shortest path length. At the worst case, it is only 1.25 times the length. In 40% percent of all samples, the hop length of the third-shortest path is the same as the hop length of the shortest path. In the worst case, it is only 1.25 times the length. Thus, more detours exist in the dense case. However they lie in a narrow area near the shortest path. As a result, the path candidates are simultaneously affected by environmental fluctuation.

From the above results, the path candidates consist of paths that take a roundabout route when the node density *d* is small, such as when *d* = 2. In such cases, each path is not simultaneously affected by environmental fluctuation. However, the alternate paths are longer than the shortest path. In contrast, when the node density *d* is large, such as when *d* = 15, the path candidates include paths of equal length, but these paths are apt to be simultaneously affected by environmental fluctuation.

In the next subsection, we investigate an appropriate design for multipath routing that depends on the degree of environmental fluctuation *q*_max⁡_.

### 3.3. Appropriate Design Depending on Degree of Fluctuation

We discuss an appropriate design for multipath routing depending on the degree *q*_max⁡_ of environmental fluctuation. For this purpose, we conduct simulation experiments with three designs that have different levels of optimality and robustness (as defined in Section 2.2) and examine different node densities *d*. The source node can avoid selecting a path susceptible to environmental fluctuation by making the robustness higher. However, it becomes difficult for the source node to select the shortest path when its optimality is lower, even when the path would not be affected by environmental fluctuation.

Rule-A maximizes optimality without concern for robustness. For the rule, probabilities *p*_1_, *p*_2_, and *p*_3_ are set at 1, 0, and 0, respectively. Rule-B lowers the optimality from Rule-A but raises the robustness. For the rule, probabilities *p*_1_, *p*_2_, and *p*_3_ are set at 0.7, 0.15, and 0.15, respectively. Rule-C further lowers the optimality and further raises the robustness from Rule-B. For the rule, probabilities *p*_1_, *p*_2_, and *p*_3_ are set at 0.4, 0.3, and 0.3, respectively. In all cases, the constant *σ* ^2^ is 1, the node density *d* is 2 or 15, and the degree *q*_max⁡_ of environmental fluctuation ranges from 0 to 1 in step sizes of 0.1.

**Figure 3:**
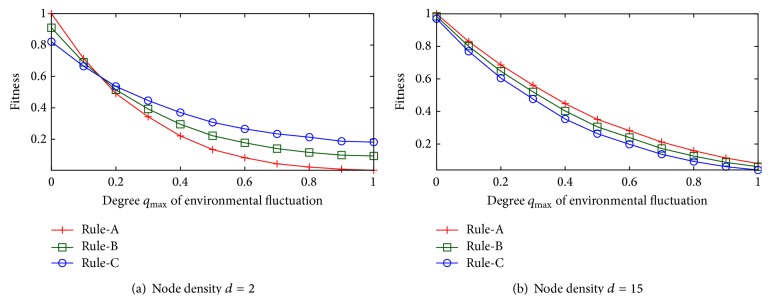
Impact of environmental fluctuation on the average fitness.

Figure 3 shows the simulation results. In the figure, the *x*-axis corresponds to the degree *q*_max⁡_ of environmental fluctuation, and the *y*-axis corresponds to fitness. Here, fitness is defined as *n*_1_/*n*_*j*_ when the destination node succeeds in receiving a packet using path *j* and is defined as 0 otherwise. We choose topologies with 3 or more node-disjoint paths. We send 1,000 packets, and we calculate the average fitness by using the 1,000 generated samples. For the sake of simplicity, we use the term “fitness” for the average of fitness as defined above.

First, we investigate the case with *d* = 2 where paths are not simultaneously affected by environmental fluctuation but the lower-ranked paths are longer than the shortest path.  Figure 3(a) shows that Rule-A is the most effective to obtain the highest fitness when the degree *q*_max⁡_ of environmental fluctuation is near 0. By prioritizing increased optimality, Rule-A sacrifices its robustness. This leads to difficulty in maintaining fitness as the degree of fluctuation *q*_max⁡_ increases. At a certain point, the fitness achieved under Rule-A is exceeded by the fitness under Rule-B and Rule-C. Specifically, when the degree *q*_max⁡_ is about 0.15, the fitness under Rule-A drops to the fitness achieved with under other rules. As the degree of *q*_max⁡_ becomes higher, the difference becomes much clearer.

Next, we investigate the case when *d* = 15 where path candidates are equally short but the path candidates are apt to be simultaneously affected by environmental fluctuation.  Figure 3(b) shows that a higher degree *q*_max⁡_ leads to the deterioration of fitness, regardless of the chosen rule, but Rule-A always results in the highest fitness. This is because the path candidates are spatially close to each other, and so they are apt to be simultaneously affected by environmental fluctuation. Thus, each path is disconnected at a similar rate. In such cases, the path candidates are prepared just only considering the increase in the optimality. These cases do not produce an effect of the disordering energy. As a result, an increase in the robustness does not contribute to keeping good fitness against an increase in the degree of fluctuation *q*_max⁡_. The detail about this will be discussed in the next section, but it is here better to prioritize optimality, that is, ensuring that the source node selects the shortest path. Rule-A, which has the highest optimality, therefore succeeds in obtaining the highest fitness.

These results imply that we should design multipath routing so that higher robustness is sought when the path candidates include paths insensitive to environmental fluctuation and paths that are likely to work in a more frequently fluctuating environment. In the next section, we establish a design policy for network control from the perspective of thermodynamic free energy.

## 4. Free-Energy-Based Design Policy

We establish an appropriate policy for designing a network control system that balances optimality against robustness according to the degree of environmental fluctuation, which we assume will occur. For this purpose, we first abstract the features of network control. Next, we build an analytical model. Then, we formulate that analytical model by a free-energy model. Finally, we analyze the degree of free energy and construct a design policy for the network control.

### 4.1. Abstraction of Network Control

**Figure 4:**
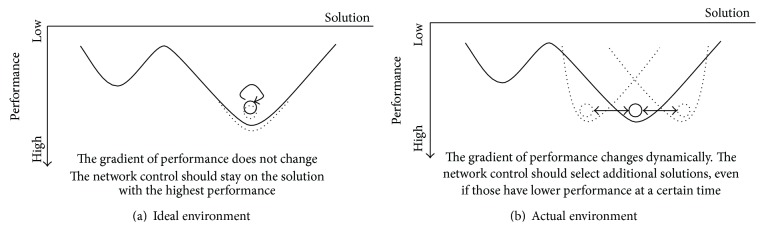
Abstract image of bioinspired network controls.

Figure 4 is an abstract image of a network control. In the figure, the *x*-axis corresponds to solutions, which the network control can select for discovering a better solution, and the *y*-axis indicates the performance when the network control selects the corresponding solution. Each black circle denotes a state of the network control at a certain time. Taking multipath routing as a network control, solution *x* corresponds to a path, and its performance corresponds to the shortness of the path. A solution affected by environmental fluctuation corresponds to a path disconnected by link errors, node failures, and so on.

The network control eventually stabilizes on the solution with the highest performance at a certain time. In ideal environments without fluctuation, the gradient of performance does not change. When this is the case, it is the best action for the network control to stay on the solution with the highest performance, as shown in Figure 4(a). However, actual networks are typically affected by environmental fluctuation, and so the gradient of performance changes dynamically. To suppress the influence of environmental fluctuation, the network control must avoid stabilizing on the solution with the highest performance at a particular moment in time. It is important that the network control selects additional solutions, even if those have lower performance at a certain time, as shown in Figure 4(b).

### 4.2. Analytical Model of Abstract Network Control

We describe an analytical model of the abstract network control. In this analytical model, the performance of solution *x* is given by a Gaussian function *g*(*x*) = *g*_max⁡_ × exp⁡[−*x* ^2^/2*σ*_2_^2^] when solution *x* is not affected by environmental fluctuation. The performance is 0 when solution *x* is affected by environmental fluctuation. Here, the coefficient *g*_max⁡_  (0 ≤ *g*_max⁡_ ≤ 1) sets a maximum value for the performance. Solution *x* is affected by environmental fluctuation per unit time with probability *q*(*x*) = *q*_max⁡_ × exp⁡[−*x* ^2^/2*σ*_3_^2^]. Thus, solution *x* is, on average, affected by environmental fluctuation at intervals of exp⁡[*x* ^2^/2*σ*_3_^2^]/*q*_max⁡_. Here, coefficient *q*_max⁡_  (0 ≤ *q*_max⁡_ ≤ 1) indicates the maximum degree of environmental fluctuation. Solution *x* is selected with probability $p(x)=\exp[-x^{2}/2\sigma_{1}^{2}]/\sqrt{2\pi \sigma_{1}^{2}}$. The descriptions of these parameters are summarized in Table 3.

### 4.3. Definition of Free Energy of Analytical Model

In Section 2, we stated that, in a fluctuation environment, the free energy *A* is identical to performance *G* that is actually achieved by the network control. For the analytical model, performance *G* can be formulated as the following equation:

(3) G=∫−∞∞12πσ12×e−x2/2σ12×gmax⁡×e−x2/2σ22×1−qmax⁡×e−x2/2σ32+0×qmax⁡×e−x2/2σ32dx=gmax⁡×σ22σ22+σ12−qmax⁡×gmax⁡×σ22σ32σ22σ32+σ12σ22+σ32.

Here, the first term on the right side is the maximum performance that can be achieved in an environment without fluctuation. This term corresponds to optimality. The second term indicates performance degradation due to environmental fluctuation. This term corresponds to robustness (see Section 2.2 for the precise definitions of optimality and robustness). The aim of this paper is to present an appropriate design for a network control that accounts for the degree of environmental fluctuation, with the intent of preparing the network control for fluctuation. To meet this objective, we regard the coefficient *q*_max⁡_ as the expected degree of environmental fluctuation. In the following, we discuss a design appropriate for a network control that accounts for environmental fluctuation.

### 4.4. Design Policy Depending on Degree of Fluctuation

Maximizing performance *G* is identical to balancing the robustness, which corresponds to the second term, with the optimality, which corresponds to the first term, for a given degree *q*_max⁡_ of environmental fluctuation. To achieve this, we first describe how to maximize the optimality. Then, we describe how to maximize the robustness. Finally, we consider an appropriate balance between the optimality and the robustness according to the expected degree *q*_max⁡_ of environmental fluctuation.

*(i) For Maximizing Optimality*. The first term on the right side of (3) indicates the maximum value of performance *G* and is identical to the performance achieved by the network control in an environment without fluctuation. Therefore, we adjust variance *σ*_1_^2^ depending on the path candidates, which depend on variance *σ*_2_^2^, so as to maximize the optimality. To do this, variance *σ*_1_^2^ must fall when variance *σ*_2_^2^ does. This suggests that the network control will more aggressively select solutions with higher performance as the solution candidates include more solutions with smaller performance.

*(ii) For Maximizing Robustness*. The second term on the right side of (3) indicates the degradation of performance *G* due to environmental fluctuation. Larger values for this term imply weaker robustness against environmental fluctuation. Therefore, to maximize the robustness, we need to minimize the second term for the solution candidates by parameter tuning. To minimize the second term, we reduce variance *σ*_2_^2^ as variance *σ*_3_^2^ grows. If we cannot reduce *σ*_2_^2^ due to restrictions on the network environment (e.g., such as those discussed for the network topology and node density *d* in Section 3), then *σ*_1_^2^ should be made larger. These relations suggest that we should prepare solution candidates having lower performance because the influence of environmental fluctuation will be relatively suppressed in those solutions. Furthermore, when it is not possible to prepare these solutions, it is better for the network control to increase its randomness in selecting solutions so that selecting a solution susceptible to environmental fluctuation becomes less likely.

*(iii) For Balancing Robustness with Optimality*. We regard (3) as a one-dimensional function of degree *q*_max⁡_ of environmental fluctuation. Then, we expect that an appropriate balance between the robustness and the optimality exists and depends on degree *q*_max⁡_. As examples, when degree *q*_max⁡_ is small, such as when *q*_max⁡_ = 0, it is more effective to prioritize maximizing optimality, that is, to make variance *σ*_1_^2^ smaller. When degree *q*_max⁡_ is large, such as when *q*_max⁡_ = 1, it is more effective to prioritize maximizing robustness, that is, to make variance *σ*_1_^2^ larger. These cases imply that we must appropriately choose a balance between optimality and robustness, and this balance will depend on the expected degree of environmental fluctuation.

**Figure 5:**
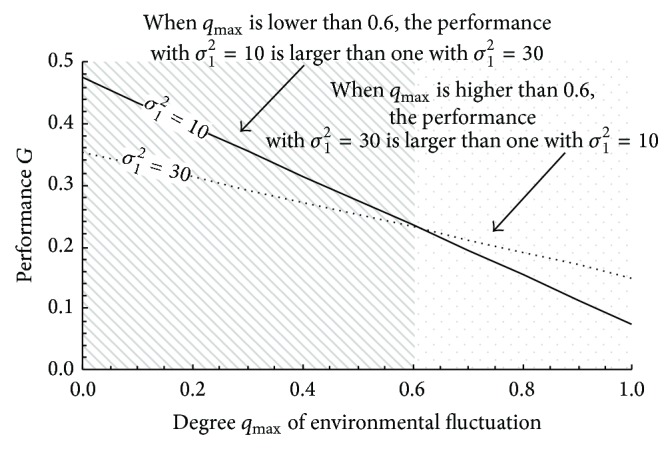
Tradeoff between robustness and optimality.

Using numerical examples, we first show that degree *q*_max⁡_ of environmental fluctuation affects which the design should prioritize, maximizing optimality or maximizing robustness.  Figure 5 shows the numerical examples. In the figure, the *x*-axis corresponds to the degree *q*_max⁡_ of environmental fluctuation, and the *y*-axis corresponds to performance *G* as obtained from (3). Here, degree *g*_max⁡_, variance *σ*_2_^2^, and variance *σ*_3_^2^ are set at 0.5, 30, and 15, respectively. As the parameter setting for the optimality to be prioritized, variance *σ*_1_^2^ is set at 10. As a parameter setting for the robustness to be prioritized, variance *σ*_1_^2^ is set at 30. The figure depicts the results. From this figure, we see that the robustness should be prioritized when *q*_max⁡_ > 0.6. This shows that the appropriate balance depends on degree *q*_max⁡_ of environmental fluctuation.

**Figure 6:**
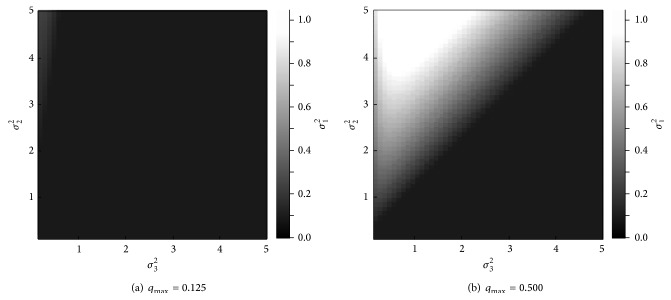
Optimal value of variance *σ*_1_^2^.

**Figure 7:**
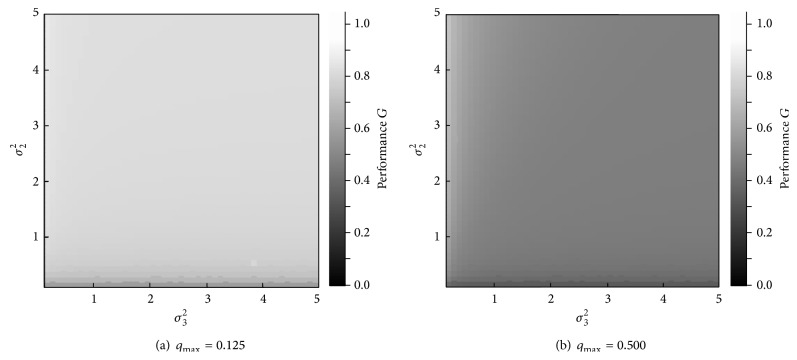
Optimal value of the performance *G*.

Next, we investigate the appropriate balance according to degree *q*_max⁡_ of environmental fluctuation and the solution candidates. For this purpose, we numerically derive variance *σ*_1_^2^ at which performance *G* is maximized when parameters *σ*_2_^2^, *σ*_3_^2^, *g*_max⁡_, and *q*_max⁡_ are fixed. For the testing, variances *σ*_2_^2^ and *σ*_3_^2^ range from 0.1 to 5.0 in steps of 0.1. Coefficient *g*_max⁡_ is taken as 1, and coefficient *q*_max⁡_ is taken as 0.125 or 0.5. Altogether, this results in 5,000 sets of parameter values.  Figure 6 shows the results. Similarly, the results for the maximum performance *G* are shown in Figure 7. In each figure, the *x*-axis is variance *σ*_3_^2^, and the *y*-axis is variance *σ*_2_^2^. Shading intensity indicates variance *σ*_1_^2^ (Figure 6) or performance *G* (Figure 7). In each figure, lighter shades indicate higher values.

Figure 6(a) shows that shading for a wide range of gradients is darker when degree *q*_max⁡_ is set at 0.125. This result suggests that the maximization of the optimality in case of *q*_max⁡_ = 0.125 is prioritized so as to maximize performance *G*. In contrast, the shading is lighter when degree *q*_max⁡_ is 0.5, as shown in Figure 6(b). This result suggests that when *q*_max⁡_ = 0.5, robustness is more heavily prioritized. In the analytical model, the robustness is sacrificed by enhancing the optimality. To achieve a balance between robustness and optimality when *q*_max⁡_ is 0.5, it is better that variance *σ*_1_^2^ is set below the maximum value of 5; here, it is set at 1. Additionally, we can notice a trend in which the shading becomes lighter when variance *σ*_2_^2^ is higher than variance *σ*_3_^2^. This result implies that the optimality needs to be prioritized if the solution candidates include solutions that have higher performance and are not susceptible to environmental fluctuation. In contrast, we can also see a trend in which the shading becomes darker when variance *σ*_2_^2^ is less than variance *σ*_3_^2^. The solution-selection rule does not increase performance *G* when almost all solution candidates are susceptible to environmental fluctuation. In this case, it is better to prioritize optimality in order to maximize performance *G*, such as Rule-A in Figure 3(b).

## 5. Verification of Design Policy

We verify the free-energy-based design policy for network controls. For this purpose, we introduce an existing system for multipath routing and identify the parameter that changes its balance between optimality and robustness. Then, we investigate the appropriate balance between the characteristics. Finally, we verify the design policy described in the previous section.

### 5.1. Example of Existing Multipath Routing

We take an attractor selection model for multipath routing [9, 12] as an example of an existing system for multipath routing.

#### 5.1.1. Mechanism of Path Selection

We make two assumptions. The first is that *K* node-disjoint paths are constructed between a source node and a destination node in advance. The second is that the source node knows the addresses of all wireless nodes on all node-disjoint paths. A path *j* ∈ {1,2,…, *K*} has a state value *m*_*j*_  (≥0). At an interval of *I*_*c*_, the source node selects the path with the maximum state value from among all paths. The state value *m*_*j*_ evolves according to the following equation:

(4) $$\begin{array}{c} {\dot{m}}_{i}=\left(\;\frac{\beta \times \alpha^{\gamma }+1/\sqrt{2}}{1+\max_{1\leq j\leq K}m_{j}^{2}-m_{i}^{2}}-m_{i}\;\right)\times \alpha +\eta_{i}. \end{array}$$

Here, there are *K* attractors that depend on which of the state values is the largest. Coefficient *β*  (>0) sets the maximum depth of attractors, and coefficient *γ*  (>0) sets the magnitude of attraction by attractors. The term *η*_*i*_ is a random value with mean 0 and variance 1 (i.e., stochastic noise). The variable *α*  (0 ≤ *α* ≤ 1) is the goodness of the multipath routing. The details are described later, but, broadly, variable *α* becomes larger as the source node more stably selects a shorter and better connected path. We hereinafter call this variable “activity.”

#### 5.1.2. Derivation of Activity

The source node calculates the activity *α* based on the path length selected by the source node and its connectivity.

To obtain a path's connectivity, the source node observes its connectivity in a periodic manner. For this purpose, the source node sends an “observation packet” at intervals of *I*_*s*_ along the path selected by the above-mentioned mechanism of path selection. In each observation packet, the source node stores a list of the addresses of wireless nodes on the path. The relay nodes iteratively forward the packet by following the list. When the destination node receives the packet, the destination node sends a “notification packet” back to the source node. This notification packet is iteratively forwarded along the reversed path of the corresponding observation packet. If the source node does not receive the notification message before it sends the next observation packet, the source node assumes that the path was disconnected by environmental fluctuation. Even if the source node later receives the notification packet, the packet will be dropped.

From the results of observation, the source node updates the activity *α* at an interval of *I*_*a*_ as follows:

(5) $$\begin{array}{c} \dot{\alpha }=\delta \times \left(\;\frac{L_{\min}}{L_{\text{now}}}-\alpha \;\right). \end{array}$$

In this equation, coefficient *δ* is a smoothing coefficient; *L*_min⁡_  (>0) is the minimum hop length of connected paths within the last *I*_*h*_ observations; and *L*_now_  (>0) is the hop length of the last-observed path. Note here that *L*_min⁡_/*L*_now_ is 0 when the last-observed path was not connected.

#### 5.1.3. Behavior of Multipath Routing

The source node sends a “data packet” at intervals of *I*_*d*_s along the selected path, which is determined by the attractor selection mechanism. As the source node selects a worse path, such as a longer path or a disconnected path, the activity *α* gradually decreases. Simultaneously, the relative influence of the noise term *η*_*i*_ in (4) on the change of the state value *m*_*i*_ becomes larger. The magnitude relationship of the state values is apt to be changed by the noise term, and so the source node selects a path more randomly in its search for a better path. As the source node finds better paths, the activity *α* gradually becomes larger. Simultaneously, the relative influence of the first term of (4) on the change of the state value *m*_*i*_ becomes larger. The state value of the found path increases, and the other state values decrease. Eventually, the magnitude relationship of the state values becomes stable, and the source node selects a specific path more stably.

#### 5.1.4. Parameter for Balance between Robustness and Optimality

In the attractor selection model of multipath routing, parameter *β* affects the balance between optimality and robustness. Specifically, as parameter *β* becomes smaller, the source node becomes more likely to stabilize on only the shortest path. Therefore, we can regard the maximization of optimality as the minimization of parameter *β*. In the other direction, as parameter *β* becomes larger, the source node becomes more likely to stabilize on a path without regard to its hop length, increasing the chance that communication between the source node and the destination node will be maintained. This is because the source node can easily stabilize on connected paths regardless of hop length. Thus, we can regard the maximization of robustness as the maximization of parameter *β*. In the following, we therefore call parameter *β* the “balance parameter.”

### 5.2. Simulation Experiment

We verify the free-energy-based design policy through simulation experiments using the attractor selection model of multipath routing. For this purpose, we derive an appropriate balance between robustness and optimality by investigating the approach-balancing parameter *β*. Comparing the results obtained by simulation with those described in Section 4.4, we verify the design policy.

#### 5.2.1. Simulation Setting

Using the simple model of a multipath network described in Section 3, we prepare a wireless network and path candidates. The parameter values are set as follows. We evaluate two node densities, *d* = 2 and *d* = 15. In the case of node density *d* = 2, there are path candidates that are not simultaneously affected by environmental fluctuation, but almost all of the path candidates are longer than the shortest path. In the other case (*d* = 15), there are path candidates as short as the shortest path, but the path candidates are simultaneously affected by environmental fluctuation. Degree *q*_max⁡_ of environmental fluctuation ranges from 0.0 to 0.5 at intervals of 0.125. Variance *σ* ^2^ is set at 1. The balance parameter *β* ranges from 5 to 20 at intervals of 5. As the balance parameter *β* becomes larger, the maximization of the robustness becomes more prioritized. The other parameters are set as shown in Table 4. The initial state vector $\vec{m}$ is set at $\vec{m}=(\beta ,0,0)$. The initial activity *α* is set at 1. In this parameter setting, the shortest path is stably selected by the source node just after a simulation begins. The following simulation results are the average values from across 500 simulation runs, where the duration of a single run is 1,000 s.

#### 5.2.2. Simulation Result

**Figure 8:**
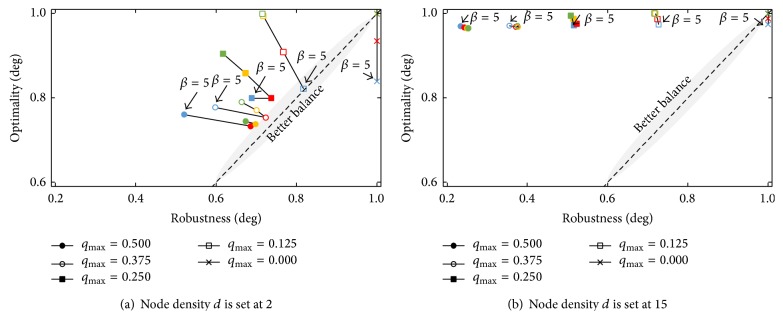
Influence of balance parameter *β* of attractor selection model on balance between robustness and optimality.

Figure 8 shows the results. In the figure, the *x*-axis corresponds to the ratio of the number of data packets received by the destination node relative to the number of data packets sent by the source node during a simulation run. This ratio measures the robustness, which here means the degree to which communication is maintained despite environmental fluctuation. We hereinafter call this ratio the “degree of robustness.” The *y*-axis corresponds to the relative length of the path used to send the data packet to the destination node. The relative length is defined as *n*_*j*_/*n*_1_, where *n*_*j*_ is the hop length of path *j* and path 1 is the shortest path. This value corresponds to the optimality, which measures the quality of communication. This value is hereinafter called the “degree of optimality.” The balance between optimality and robustness is taken as better when the degree of robustness is closer to the degree of optimality and both are higher. The balance parameter *β* is distinguished in the figure by symbol color. The blue, red, orange, and green symbols denote results for *β* = 5, 10, 15, and 20, respectively.

First, we discuss the simulation results when we can obtain path candidates that are not simultaneously affected by environmental fluctuation, although they include longer paths.  Figure 8(a) shows that it is better that optimality is more prioritized when environmental fluctuation occurs less frequently. From this figure, we can see that a parameter value of *β* = 15 achieves the best balance between optimality and robustness when degree *q*_max⁡_ of environmental fluctuation is 0.5. This result agrees with the suggestion that the maximization of robustness is prioritized; that is, the balance parameter *β* is best set at larger values when environmental fluctuation occurs more frequently. However, it is not always better to increase *β*. In this simulation setting, the best value for *β* is not the maximum value of 20; it is 15. As degree *q*_max⁡_ becomes smaller than 0.5, the best value for balance parameter *β* becomes smaller. This implies that it is good to prioritize optimality more strongly as degree *q*_max⁡_ becomes smaller. Here, when degree *q*_max⁡_ is set at 0.375 and 0.25, setting the parameter *β* to 10 achieves the best balance. In addition, setting *β* to 5 achieves the best balance when degree *q*_max⁡_ is set at 0.125. However, there is an inconsistency in this figure. The best value for *β* is 20 when degree *q*_max⁡_ is set at 0, that is, when environmental fluctuation does not occur. In this simulation setting, just after the simulation begins, the source nodes stably select the shortest path. When degree *q*_max⁡_ is set at 0, the shortest path is always connected. In this case, the best balance between optimality and robustness is achieved by the source node continuing to select the shortest path until the simulation finishes. Thus, the best value for *β* is 20, the maximum possible value in this simulation setting; however, this depends on the initial setting of the simulation.

Next, we discuss the simulation result when we can obtain a set of path candidates that include equally short paths as the shortest path but candidates are simultaneously affected by environmental fluctuation. The simulation results are shown in Figure 8(b). This figure shows that the value of the balance parameter *β* does not affect the balance between optimality and robustness, and so no single appropriate balance exists. When the node density *d* is 15, the path candidates lie in a narrow area near the shortest path, and each path is disconnected with similar frequency. A rule for path selection therefore does not contribute to maintaining communication between the source node and the destination node. That is, we cannot improve robustness by adjusting the balance parameter *β*. Therefore, there is no best balance between robustness and optimality.

In conclusion, the balance parameter *β* should be set at a larger value for the network control to be equipped with the ability to endure more frequent environmental fluctuation, but this is helpful only when the path candidates include paths not simultaneously affected by environmental fluctuation. This suggestion agrees with the free-energy-based design policy described in the previous section.

## 6. Related Work

Researchers are focusing on network controls based on biological self-organization (hereinafter, BSON) in order to realize more robust communication networks. For instance, ant colony optimization [13], which is inspired by the foraging behavior of ants, has been applied to routing algorithms [14], pulse-coupled oscillation [15], which is inspired by the synchronization behavior of fireflies, has been applied to time synchronization algorithms [16], and response threshold model [5], which is inspired by the division of labors in social insects, has been applied to adaptive task allocation mechanism [17]. Useful global behaviors arise from direct and indirect interactions among nodes. However, this bottom-up approach can lead to difficulty in designing or optimizing the global behaviors of BSON controls.

To optimize a BSON control, many researchers analyze the influence of its control parameters on its characteristics (such as optimality and robustness) through simulation mathematical analysis [18, 19]. However, each analysis is typically focused on a specific BSON control. As a consequence, there is little general knowledge that can be used in optimizing existing or future BSON controls. Some other studies have tried to quantitatively define characteristic values (see, e.g., [20, 21]). Such general definitions are helpful during the design and optimization of existing and future BSON controls. However, it is typically supposed that each element's behavior can be represented by a simple stochastic automaton. This makes it difficult to define the characteristic values of a BSON control that has complicated behaviors. As another approach, some argue that the system should be constricted in a top-down manner to realize the desired behaviors of BSON controls. For this style of approach, the self-organizing behavior is elucidated by focusing on an underlying thermodynamic principle [22, 23]. However, that work does not provide us with a concrete method for designing and optimizing BSON controls. Still other researchers have focused on directly controlling BSONs [24–26], aiming to achieve the desired behavior by controlling a part of the elements. The research along this vein, however, targets improvements in transient characteristics, such as convergence speed. It does not consider how steady states should emerge to achieve sufficient robustness and performance under a given set of network conditions.

In conclusion, there is no general method to design or optimize a BSON control while taking into account the assumed network conditions.

## 7. Conclusion and Future Work

We formulated and analyzed a free-energy model of network control. Then, we established a design policy based on the analytical results. The obtained policy suggests that network control should be designed to improve its robustness in cases where it is deployed in more dynamic environments and has solution candidates that are insusceptible to environmental fluctuation.

As future work, we intend to propose a method for deciding the value of the balance parameter *β* according to the given degree of environmental fluctuation. For this objective, we will extend the analytical model in this paper to an analytical model that extends the attractor selection model. Then, we will formulate and analyze its free energy.

## Figures and Tables

**Table 1:** Interpretation from the thermodynamic perspective.

	Description in terms of thermodynamics
Internal energy *E*	Energy that can potentially be extracted
Entropy *S*	Difficulty of extracting energy
Temperature *T*	Average energy of particles
Free energy *A*	Energy that can actually be extracted

**Table 2:** Interpretation from the perspective of network controls.

	Description in terms of network controls
Internal energy *E*_nw_	Performance that can potentially be obtained
Entropy *S*_nw_	Difficulty of maintaining performance
Temperature *T*_nw_	Frequency of environmental fluctuation
Free energy *A*_nw_	Performance that can actually be obtained

**Table 3:** Parameters of analytical model.

Symbol	Description
*q*_max⁡_	Maximum degree of environmental fluctuation
*g*_max⁡_	Maximum performance of solutions
σ_1_^2^	Randomness in selecting a solution
σ_2_^2^	Abundance of good solutions
σ_3_^2^	Extent of influence of environmental fluctuation

**Table 4:** Parameter setting.

Symbol	Value	Description
*I*_*c*_	1 s	Interval for path selection
*I*_*s*_	1 s	Interval for observing connectivity
*I*_*a*_	1 s	Interval for updating activity
*I*_*d*_	1 ms	Interval for sending of data packets
*I*_*h*_	10 packets	Length of history
γ	1.0	Magnitude of attraction
δ	0.1	Smoothing coefficient
